# MicroRNA-148b secreted by bovine oviductal extracellular vesicles enhance embryo quality through BPM/TGF-beta pathway

**DOI:** 10.1186/s40659-024-00488-z

**Published:** 2024-03-23

**Authors:** Karina Cañón-Beltrán, Yulia N Cajas, Vasileios Almpanis, Sandra Guisado Egido, Alfonso Gutierrez-Adan, Encina M González, Dimitrios Rizos

**Affiliations:** 1 Department of Biochemistry and Molecular Biology, Veterinary Faculty, Complutense University of Madrid (UCM), Madrid, Spain; 2 Programa de Medicina Veterinaria y Zootecnia, Corporación Universitaria del Huila (CORHUILA), Grupo Kyron, Huila, Colombia; 3 Department Agrarian Production, Technical University of Madrid (UPM), Madrid, Spain; 4 Departamento de Ciencias Biológicas, Universidad Técnica Particular de Loja (UTPL), Loja, Ecuador; 5 Department of Animal Reproduction, National Institute for Agriculture and Food Research and Technology (CSIC-INIA), Madrid, Spain; 6 Department of Anatomy and Embryology, Veterinary Faculty, Complutense University of Madrid (UCM), Madrid, Spain

**Keywords:** Cattle, Embryos, miRNAs, miR-148b, TGF-beta

## Abstract

**Background:**

Extracellular vesicles (EVs) and their cargoes, including MicroRNAs (miRNAs) play a crucial role in cell-to-cell communication. We previously demonstrated the upregulation of bta-mir-148b in EVs from oviductal fluid of cyclic cows. This miRNA is linked to the TGF-β pathway in the cell proliferation. Our aim was to verify whether miR-148b is taken up by embryos through gymnosis, validate its target genes, and investigate the effect of miR-148b supplementation on early embryo development and quality.

**Methods:**

Zygotes were cultured in SOF + 0.3% BSA (Control) or supplemented with: 1 µM miR-148b mimics during: D1-D7 (miR148b) or D1-D4 (miR148b-OV: representing miRNA effect in the oviduct) or D4-D7 (miR148b-UT: representing miRNA effect in the uterus) or 1 µM control mimics was used during: D1-D7 (CMimic). Embryos at ≥ 16-cells and D7 blastocysts (BD7) were collected to examine the mRNA abundance of transcripts linked to the TGF-β pathway (*TGFBR2, SMAD1, SMAD2, SMAD3, SMAD5, BMPR2, RPS6KB1, POU5F1, NANOG)*, total cell number (TC), trophectoderm (TE), and inner cell mass (ICM) were also evaluated. One-way ANOVA was used for all analyses.

**Results:**

We demonstrated that miR-148b can be taken up in both 16-cell embryos and BD7 by gymnosis, and we observed a decrease in *SMAD5* mRNA, suggesting it’s a potential target of miR-148b. Cleavage and blastocysts rates were not affected in any groups; however, supplementation of miR-148b mimics had a positive effect on TC, TE and ICM, with values of 136.4 ± 1.6, 92.5 ± 0.9, 43.9 ± 1.3 for miR148b and 135.3 ± 1.5, 92.6 ± 1.2, 42.7 ± 0.8, for miR148b-OV group. Furthermore, mRNA transcripts of SMAD1 and SMAD5 were decreased (*P* ≤ 0.001) in 16-cell embryos and BD7 from miR148b and miR148b-OV groups, while *POU5F1* and *NANOG* were upregulated (*P* ≤ 0.001) in BD7 and *TGFBR2* was only downregulated in 16-cell embryos. pSMAD1/5 levels were higher in the miR148b and miR148b-OV groups.

**Conclusions:**

Our findings suggest that supplementation of bta-miR-148b mimics during the entire culture period (D1 - D7) or from D1 - D4 improves embryo quality and influences the TGF-β signaling pathway by altering the transcription of genes associated with cellular differentiation and proliferation. This highlights the importance of miR-148b on embryo quality and development.

**Supplementary Information:**

The online version contains supplementary material available at 10.1186/s40659-024-00488-z.

## Introduction

Different approaches have been used to understand the mechanisms of the oviduct’s contribution to early reproductive events, such as early embryo development [1]. Initially, it was believed that indirect intercellular communication occurred through the transfer of secreted molecules called embryotropins [2]. However, in recent years, extracellular vesicles (EVs), membrane-limited vesicles found in the extracellular environment, have gained interest in relation to communication between the embryo and the oviduct [3]. Studies have demonstrated that EVs can be both secreted and received by the bovine embryo [4, 5], as well as secreted by the oviduct [6, 7] and uterus [8, 9], suggesting bidirectional embryo-maternal communication. Furthermore, in vitro studies have confirmed that preimplantation embryos communicate with the oviduct [10] and uterus [11, 12].

EVs mediate cell-to-cell communication by transferring biomolecules, such as microRNAs (miRNAs), which can modulate the activities of recipient cells. MiRNAs are small, natural, single-stranded non-coding RNA molecules of 20–24 nucleotides that play a crucial role in almost all biological processes in multicellular organisms, especially in mammals [13–15]. MiRNAs bind to their target mRNAs, leading to translational repression and mRNA degradation [16]. As complete complementarity for binding is not required for miRNAs to function, a single miRNA can downregulate the production of various proteins and target genes [17]. The membrane of EVs plays a crucial role in effectively shielding the enclosed cargo contents (miRNAs, mRNAs) from RNase activity in the surrounding medium. This protective mechanism contributes to the enhanced stability and reliability observed in miRNAs derived from EVs compared to those freely circulating [18, 19].

MiRNAs have great potential as biomarkers for a wide range of applications, including disease diagnosis [20, 21], embryo development [22], assessment of preimplantation developmental competence in cattle [23], and evaluation of embryo viability in humans [24]. In a recent study by our group, the impact of EVs derived from oviductal fluid (OF) at the early luteal phase and uterine fluid (UF) at the mid-luteal phase in sequential in vitro culture (mimicking in vivo conditions) on the development and quality of bovine embryos was investigated [25]. The analysis revealed significant differences in the expression of miRNAs between EVs derived from OF and UF. Among the 20 differentially expressed miRNAs (*P* < 0.05), 19 were found to be more abundant in EVs from UF, while only one miRNA (bta-miR-148b) was more abundant in EVs from OF [25]. These findings highlight the influence of EVs origin on miRNA profiles and further emphasize the potential of miRNAs as valuable biomarkers across different fields of research and clinical applications.

Bta-miR-148b shares 100% homology with human miR-148b-3p, which has been found to be significantly downregulated in human tumor cells and associated with the suppression of cell proliferation, migration, and invasion [26]. Conversely, overexpression of miR-148b-3p has been reported to promote proliferation [27]. A study investigating the functional mechanisms of miR-148b-3p identified 405 target genes predicted by at least four algorithms. Pathway enrichment analyses revealed the involvement of these candidate miRNA targets in various pathways, including the transforming growth factor (TGF)-β signaling pathway, among others [28]. These findings were corroborated by another study, which revealed a strong enrichment of miR-148b-3p targets among the components of the TGF-β pathway. Specifically, miR-148b-3p was found to target TGFB2 and SMAD2, thereby regulating angiogenesis and endothelial-to-mesenchymal transition during the process of skin wound healing [29].

The TGF-β superfamily of growth factors includes more than 30 structurally related mammalian proteins that have diverse functions during embryonic development and adult tissue homeostasis [30]. They can be grouped into three families: the TGF-β family, the activin family, and the bone morphogenetic protein (BMP) family [30]. A prominent role for the BMP/TGF-β pathway in regulating the patterning of early embryos has been described [31]. Furthermore, BMPs have been implicated in the regulation of trophoblast differentiation in human embryonic stem cells [32] and bovine embryo-oviduct interaction [33].

Considering this evidence, the BMP/TGF-β signaling pathway could be a candidate pathway for embryo-maternal communication during the preimplantation period through miR-148b. Thus, the objectives of this study were (i) to verify the uptake of miR-148b in bovine embryos by passive transfection (gymnosis), (ii) validate its target genes in bovine embryos produced in vitro, and (iii) evaluate the effect of miR-148b supplementation on bovine in vitro early embryo development and quality and determine the relative expression of selected genes associated with the TGF-β pathway.

## Materials and methods

### Experiment design

#### Experiment 1: internalization of miR-148b in bovine embryos by passive transfection (gymnosis)

The process in which all substances can effectively be taken up by cells in the absence of any transfection vehicles is called gymnosis. So, the rationale behind this experiment was to investigate whether miR-148b mimic supplementation in the culture medium can effectively be taken up the embryos by gymnosis. For this experiment, presumptive zygotes were cultured in SOF + 0.3% BSA alone (Control, *n* = 575) or supplemented either with 1 µM miR-148b mimic (miR148b, *n* = 656); or with 1 µM control mimics (CMimic, *n* = 556) (Supplementary Fig. 1A). The developmental parameters were calculated as: (I) cleavage rate at 48 hpi; (II) developmental rate at 96 hpi: percentage of embryos that developed to the 16-cells stage; and (III) blastocyst yield: percentage of presumptive zygotes that developed to the blastocyst stage at Days 7 and 8. To confirm miRNA uptake, a representative number of 16-cell embryos on day 4 and blastocysts on day 7 from each experimental group were either: (i) fixed and stained with Hoechst 33,342 (*n* = 15/group) and observed by widefield fluorescence microscope with structured illumination (ApoTome, Zeiss) UV-2E/C, excitation: 340e380 nm, emission: 435e485 nm) to check that commercially available triple-RNA-strand mimics could pass through the zona pellucida of bovine embryos; or (ii) frozen in liquid nitrogen (LN_2_; *n* = 30 per group) in groups of 10 and stored at -80 °C to examine the expression pattern of miR-148b by RT-qPCR. Furthermore, an additional set of 16-cell embryos and BD7 (three groups of 10: *n* = 30 per group) from each experimental group was frozen in liquid nitrogen (LN_2_) and stored at -80 ºC for the validation of target gene expression (See Experiment 2). Thirteen replicates for each experimental group were performed.

#### Experiment 2: target genes prediction and miR-148b validation by RT-qPCR

Target genes were identified by computational algorithms (Targetscan, Diana, PicTar, and Miranda). If a target was identified by three algorithms, it was considered likely to be a miRNA target. In addition, the KEGG database was used to map the predicted targets of microRNAs to early embryo development pathways. The putative target genes identified in this way were analyzed by RT-qPCR using three independent pools of 10 embryos per stage (16-cells and BD7) obtained from each experimental group (produced in Experiment 1) according to the procedures described below (See section: Validation of target genes of miR-148b by RT-qPCR).

#### Experiment 3: biological role of miR-148b in early embryonic development

In this experiment, the effect of miR-148b supplementation on embryo development was evaluated during the following developmental periods: (a) from presumptive zygotes to blastocyst in SOF + 0.3% BSA (Control, *n* = 1001), (b) from presumptive zygotes to blastocyst in SOF + 0.3% BSA supplemented with 1 µM control mimics (CMimic, *n* = 1011), (c) from presumptive zygotes to blastocyst in SOF + 0.3% BSA supplemented with 1 µM of miR-148b (miR148b, *n* = 1019), (d) from presumptive zygotes to 16- cells stage in SOF + 0.3% BSA supplemented with 1 µM of miR-148b (miR148b-OV: representing miRNA effect in the oviduct, *n* = 1052), and (e) from 16-cell stage embryos to blastocyst in SOF + 0.3% BSA supplemented with 1 µM of miR-148b (miR148b-UT: representing miRNA effect in the uterus, *n* = 1056) (Supplementary Fig. 1B). The developmental parameters were measured as: (I) cleavage rate at 48 hpi; (II) developmental rate at 96 hpi: percentage of embryos that developed to the 16-cells stage; and (III) blastocyst yield: percentage of presumptive zygotes that developed to the blastocyst stage at Days 7 and 8.

To evaluate if miR-148b induces changes in the expression levels of genes related to embryo development and quality, three independent pools of 10 embryos per stage (16-cells and BD7) obtained from each experimental group, were used for gene expression analysis by RT-qPCR according to the procedures described above. The selected genes have been linked to TGF-β pathway and embryonic development and are essential in cell proliferation, differentiation, and embryo quality, such as Transforming growth factor-beta 2 receptor (*TGFBR2*); SMAD family member 1 (*SMAD1*); SMAD family member 2 (*SMAD2*); SMAD family member 3 (*SMAD3*); SMAD family member 5 (*SMAD5*); Bone morphogenetic protein receptor 2 (*BMPR2*); Ribosomal Protein S6 Kinase Beta-1 (*RPS6KB1*); POU Class 5 Homeobox 1 (*POU5F1*); Nanog Homeobox (*NANOG*). Additionally, BD7 were selected for (I) immunolocalization of POU5F1 and CDX2 (*n* = 30) and SMAD1/5 (*n* = 15), and (II) three replicates of 30 BD7: *n* = 90 BD7 from each group were frozen in LN_2_ for WB analysis.

## Materials

Unless stated otherwise, all reagents were purchased from Sigma-Aldrich Corporation (St Louis, MO, USA).

### Oocyte collection and maturation

Immature cumulus-oocyte complexes (COCs) were obtained by aspirating follicles (2–8 mm) from the ovaries of mature heifers and cows collected from a local abattoir. COCs (homogeneous cytoplasm and intact cumulus cells (CCs)) were selected and matured in four-well dishes (Nunc, Roskilde, Denmark) in 500 µL of maturation medium (TCM-199), supplemented with 10% (v/v) fetal calf serum (FCS) and 10 ng/mL epidermal growth factor (EGF), in groups of 50 COCs per well for 24 h at 38.5 ºC and in an atmosphere of 5% CO_2_ in air with maximum humidity.

### Sperm preparation and in vitro fertilization (IVF)

Briefly, frozen semen straws (0.25 mL) from an Asturian Valley bull previously tested for IVF were thawed at 37 ºC in a water bath for 1 min and centrifuged for 10 min at 280 × g through a gradient of 1 mL of 40% and 1 mL of 80% Bovipure (Nidacon Laboratories AB, Göthenborg, Sweden) according to the manufacturer´s instructions. The sperm pellet was isolated and washed in 3 mL of Boviwash (Nidacon Laboratories AB) by centrifugation at 280 × g for 5 min. The pellet was re-suspended in the remaining 300 µL of Boviwash. The final concentration of spermatozoa was adjusted to 1 × 10^6^ spermatozoa/mL. Gametes were co-incubated for 18–22 h in 500 µL of fertilization medium (Tyrode’s medium) with 25 mM bicarbonate, 22 mM sodium lactate, 1 mM sodium pyruvate and 6 mg/mL fatty acid-free bovine serum albumin (BSA) supplemented with 10 mg/mL heparin sodium salt (Calbiochem) in four-well dishes in groups of 50 COCs per well in an atmosphere of 5% CO_2_ in the air with maximum humidity at 38.5 ºC.

### In vitro culture (IVC) of presumptive zygotes

At approximately 21 h post-insemination (hpi), a total of 5139 presumptive zygotes were denuded of CCs by vortexing for 3 min and then cultured in groups of 25 in 25-µL droplets of culture medium (synthetic oviductal fluid (SOF); [34]) supplemented with 0.3% (v/v) BSA, 4.2 mM sodium lactate, 0.73 mM sodium pyruvate, 30 µL/mL basal medium eagle (BME) amino acids, 10 µL/mL minimum essential medium (MEM) amino acids and 1 µg/mL phenol red. Culture took place at 38.5 ºC in an atmosphere of 5% CO_2_, 5% O_2_, and 90% N_2_.

### Experiment 1: uptake of miR-148b in bovine embryos by passive transfection (gymnosis)

#### MiR-148b mimics supplementation and its Uptake

Using a previously described protocol by Pavani et al. [5], miRNA mimics (unique LNA-enhanced, triple-RNA strand), designed for mimicking mature endogenous miR-148b (has-miR-148b-3p) (miR148b), or negative control (fluorescently labeled miRNA mimics / miRCURY LNA miRNA Mimic − 5′ Fam, Product No. 339,173, Qiagen) (CMimic) were supplemented into the culture medium of presumptive zygotes with a final concentration of 1 µM. To this end, bovine embryos were produced according to the methods described above (see Sect. 2.3). In parallel, a Control (SOF + 0.3% BSA) was included by adding an equal volume of RNA-free water to the culture medium. Embryo development and quality was assessed, while 16-cells embryos on day 4 and blastocysts on day 7 (BD7) from each experimental group were frozen in liquid nitrogen (LN_2_) in three groups of 10 and stored at − 80 °C for RT-qPCR analysis (see next section). For fluorescence analysis 16-cells embryos (n = ${\sim}$ 30) and BD7 (n = ${\sim}$ 30), were washed twice in PBS and fixed in 4% paraformaldehyde (PF) for 30 min at room temperature and stained with Hoechst 33,342 (10 µg/mL) for 30 min. Finally, embryos were mounted and imaged at a Zeiss Axio Observer microscope coupled to ApoTome.2 or a fluorescence stereomicroscope (Zeiss V20).

#### **The expression pattern of miR-148b was analyzed using RT-qPCR**

Total RNA was isolated from three pools of ten 16-cells embryos and ten BD7 using the miRNeasy Mini kit (Qiagen, Germantown, USA), and reverse-transcribed using a miRCURY LNA miRNA PCR Starter Kit (Qiagen) according to the manufacturer’s instructions. RNA concentration and purity were determined by NanoDrop™ 1000 (Thermo Fisher Scientific, Massachusetts, USA) spectrophotometer. All samples had appropriate amounts of chloroform added to the QIAzol Lysis Reagent (Qiagen) and with the addition of 1.33 µL of co-precipitator GlycoBlue (Thermo Fisher Scientific) to the aqueous phase before RNA precipitation. The aqueous layer that formed after centrifugation at 12,000 × g for 15 min at 4 °C was collected and combined with 1.5 × volume 100% ethanol. Purification of microRNA and total RNA from this fraction was performed using miRNeasy Mini/Micro Kit (Qiagen #217,004/#217,084) as per manufacturer’s instructions. On-column DNase digestion was performed using the RNase-Free DNase Set (Qiagen #79,254). After the aqueous phase was collected for extraction of RNA. The solution was shaken vigorously for 15 s, incubated at room temperature for 2 min, and then centrifugated at 8000 × g for 15 s at 4 °C. The resulting aqueous layer was collected, and cDNA synthesis using the miRCURY LNA miRNA PCR Starter Kit (Qiagen #3,390,320) was performed following the manufacturer’s instructions. As endogenous references (housekeeping) we used *U6* [35, 36] and *SNORD61* (Qiagen), previously shown to be stable in bovine embryos that were quantified to normalized mRNA expression levels using geNorm [37].

A single first-strand cDNA synthesis reaction was carried out per sample using 10 ng of total RNA in a 10 µL cDNA synthesis reaction. Each 10 µL reaction was made up of the following: 5 µL 2× miRCURY SYBR Green Master Mix, 1 µL PCR primer mix, 0.95 µL nuclease-free water, 3 µL of cDNA template (diluted 1:60). The reaction was initially heat-activated at 95 °C for 2 min, followed by 40 cycles of denaturation (95 °C for 10 s) and combined annealing/extension (56 °C for 60 s). All mRNA transcripts were quantified in duplicate using a Rotorgene 6000 Real-Time Cycler (Corbett Research, Sydney, Australia). The miRNAs were only considered as detected when the number of cycles required for the fluorescent signal to cross the threshold (Ct value) was lower than 37. RT-qPCR reactions were performed in triplicate and according to the comparative CT method, the ΔCT value was determined by subtracting the mean CT value of the two housekeeping genes from the CT value of the gene of interest in the same sample. The calculation of ΔΔCT involved using the highest treatment ΔCT value (i.e. the treatment with the lowest target expression) as an arbitrary constant to subtract from all other ΔCT sample values. Fold-changes in the relative gene expression of the target were determined using the formula 2^−ΔΔCT^. The primer sequences used for RT-qPCR are listed in Table 1.

**Table 1:** Mature miRNA forward primers (used with Qiagen miScript Universal Primer as reverse primer)

	Primer sequence (5’- 3’)
miR-148b	UCAGUGCAUCACAGAACUUUGU
Control mimic	GAUGGCAUUCGAUCAGUUCUA
U6	CGCTTCACGAATTTGCGTGTCA
SNORD61	CCACTGATCTTCCGACATGA

### Experiment 2. Target genes prediction and miR-148b validation by RT-qPCR

#### miRNA-148b target genes prediction

To understand the mechanisms by which miR-148b affects embryonic development, we used computational algorithms to identify putative miR-148b targets in cattle. To increase the accuracy of the prediction, the genes that were predicted by Targetscan (http://www.targetscan.org/), Diana (http://diana.imis.athena-innovation.gr/DianaTools/), PicTar (https://pictar.mdc-berlin.de/), and Miranda (http://mirdb.org/) were selected as targets. If a target was identified by three algorithms, it was considered likely to be a miRNA target. The putative target genes identified in this way were analyzed by RT-qPCR. In addition, the KEGG database (http://www.genome.jp/kegg/tool/search_pathway.html) was used to map the predicted targets of microRNAs to early embryo development pathways.

#### Validation of target genes of miR-148b by RT-qPCR

Expression of target gene analysis was performed using 16-cell embryos and BD7 (three groups of 10: *n* = 30 per group). Poly(A) RNA was extracted using the Dynabeads mRNA Direct Extraction Kit (Ambion; Fisher Scientific Inc., Oslo, Norway) with minor modifications [38]. Immediately after poly(A) RNA extraction, reverse transcription (RT) was performed using a Moloney murine leukemia virus (MMLV) Reverse Transcriptase 1st-Strand cDNA Synthesis Kit according to the manufacturer’s instructions (Epicentre Technologies Corp, Madison, WI, USA). Tubes were heated to 70 ºC for 5 min to denature the secondary RNA structure, allowing Poly(T) random primers and Oligo dT annealing, and the RT mix was then completed by adding 0.375 mM dNTPs (Biotools, Madrid, Spain), 6.25 U RNAsin RNAse inhibitor (Promega, Madison, WI, USA), MMLV HP RT 10x reaction buffer, 5 mM DTT and 5 U MMLV high-performance reverse transcriptase (Epicentre Technologies Corp, Madison, WI, USA). Samples were incubated at 25 ºC for 10 min and then at 37 ºC for 60 min, to allow the RT of RNA, and finally at 85 ºC for 5 min to denature the enzyme. All mRNA transcripts were quantified in duplicate using a Rotorgene 6000 Real-Time Cycler (Corbett Research). RT–quantitative polymerase chain reaction (qPCR) was performed by adding a 2 µL aliquot of each cDNA sample (〜60 ng µL-1) to the PCR mix (GoTaq qPCR Master Mix, Promega) containing specific primers to amplify the genes of interest. Primer sequences are provided in Supplementary Table S1. All primers were designed using Primer-BLAST software (http://www.ncbi.nlm.nih.gov/tools/primer-blast/) to span exon-exon boundaries when possible. For quantification, RT-qPCR was performed as described previously [39]. The PCR conditions were tested to achieve efficiencies close to 1. Relative expression levels were quantified by the comparative cycle threshold (CT) method [40]. Values were normalized using two housekeeping genes: *H2AFZ* and *RN18S1*. Fluorescence was acquired in each cycle to determine the threshold cycle or the cycle during the log-linear phase of the reaction at which fluorescence increased above the background for each sample. Within this region of the amplification curve, a difference of one cycle is equivalent to a doubling of the amplified PCR product. According to the comparative CT method, the ΔCT value was determined by subtracting the mean CT value of the two housekeeping genes from the CT value of the gene of interest in the same sample. The calculation of ΔΔCT involved using the highest treatment ΔCT value (i.e. the treatment with the lowest target expression) as an arbitrary constant to subtract from all other ΔCT sample values. Fold-changes in the relative gene expression of the target were determined using the formula 2^−ΔΔCT^.

### Experiment 3: biological role of miR-148b in early embryonic development and quality

#### miR-148b supplementation during IVC

The effects of miR-148b supplementation during IVC was evaluated during the following developmental periods: (a) from presumptive zygotes to blastocyst in SOF + 0.3% BSA (Control), (b) from presumptive zygotes to blastocyst in SOF + 0.3% BSA supplemented with 1 µM control mimics (CMimic), (c) from presumptive zygotes to blastocyst in SOF + 0.3% BSA supplemented with 1 µM of miR-148b (miR148b), (d) from presumptive zygotes to 16- cells stage in SOF + 0.3% BSA supplemented with 1 µM of miR-148b (miR148b-OV: representing miRNA effect in the oviduct), and (e) from 16-cell stage embryos to blastocyst in SOF + 0.3% BSA supplemented with 1 µM of miR-148b (miR148b-UT: representing miRNA effect in the uterus) (Supplementary Fig. 1B). Bovine embryos were produced according to the methods described above (see Sect. 2.3).

A representative number BD7 from each experimental group (n = ${\sim}$ 30 per group) were stained with anti-CDX2, POU5F1 and Hoechst to evaluate the pluripotency such as formation of the trophectoderm (TE) and inner cell mass (ICM), and pSMAD1/5 to evaluate the functional specificity of miR-148b (n = ${\sim}$ 15 per group). Additionally, a representative number of 16-cell embryos and BD7 from each experimental group were frozen in liquid nitrogen (LN_2_) and stored at − 80 ◦C, for gene expression analysis (three groups of 10: *n* = 30 per group) and for western blotting (WB) analysis (three groups of 30: *n* = 90 blastocysts per group). Thirteen replicates were performed under the same assay conditions.

#### Immunofluorescence of POU5F1 and CDX2 in blastocysts

Immunolocalization of POU5F1 and CDX2 was performed according to Cajas et al. [41] with minor modifications. For this analysis, 30 BD7 from all experimental groups were washed twice with PBS + 0.1% PVP and fixed in 4% PF for 10 min at room temperature. Next, cells were permeabilized in PBS + 5% goat serum (IF buffer) with 1% Triton X-100 for 45 min at room temperature. Then the samples were incubated overnight at 4 ºC with the primary antibodies: CDX2 (1:200, Biogenix, Fremont, CA, USA), Oct-3/4 (1:200, Santa Cruz Biotechnology, sc-5279) dilution in blocking solution. Following incubation, samples were washed twice in PBS + 1% BSA and incubated in the secondary antibody solution consisting of PBS + 1% BSA 20% IF buffer, 1:3000 Alexa Fluor goat anti-mouse 488 (Invitrogen, Grand Island, NY, USA), for 2 h at room temperature followed by washing again three times in PBS + 0.1% PVP. In all cases, nuclei were stained with Hoechst 33,342 (10 µg/mL). Finally, the samples were mounted in microdrops with ProLong™ Diamond between a coverslip and a glass slide, sealed with nail polish, and analyzed by widefield fluorescence microscope with structured illumination (ApoTome, Zeiss) UV-2E/C. Images obtained were evaluated using the ImageJ program (NIH, ImageJ version 1.52 k software (http://rsbweb.nih.gov/ij/)).

#### Immunofluorescence of pSMAD1/5 in blastocysts

Immunolocalization of pSMAD1/5 was performed as described previously in Sect. 2.6.2 with minor modifications. For immunostaining, 15 blastocysts of Day 7 from all experimental groups were permeabilized in PBS with 10% FCS and 1% Triton X-100 for 45 min at room temperature. After permeabilization, the samples were incubated overnight at 4 ºC in PBS + 0.1% PVP and 5% FCS and pSMAD1/5 (1:500, Ser463/465, Catalog #9516, Cell Signaling). Following incubation, samples were washed twice in PBS + 0.1% PVP and incubated in PBS supplemented with 5% FCS and 1:250 goat anti-mouse polyclonal antibody Alexa Fluor 488-conjugate (Molecular Probes, Eugene, OR, USA), and Hoechst 33,342 (10 µg/mL) for 2 h at room temperature. Finally, the samples were mounted in microdrops with ProLong™ Diamond and analyzed by widefield fluorescence microscope with structured illumination (ApoTome, Zeiss) UV-2E/C. Negative control was prepared, and images obtained were evaluated using the ImageJ program.

#### Embryo gene expression analysis

Gene expression analysis was performed on 16-cells embryos and BD7 as described previously in Sect. 2.5.2.

#### Western blotting

Western blotting analysis was performed as described previously by Cañón-Beltrán et al. [42] with minor modifications. Day 7 blastocysts from all experimental groups were lysed in 300 µL RIPA buffer (150mM NaCl, 1% Triton X-100, 0.5% sodium deoxycholate, 0.1% SDS, and 50mM Tris [pH 7.6]), supplemented with 1× protease, phosphatase Inhibitor Cocktail (Roche, Basel, Switzerland), for 1 h at 4 ºC. The samples were mixed with 1× sample buffer, and then denatured at 95 °C for 5 min. Proteins were resolved by SDS-PAGE (12% acrylamide gel loading 45 µL of total protein per well) and transferred onto a nitrocellulose membrane for immunoblotting following standard procedures. After the transfer, membranes were blocked for 30 min in 3% BSA in PBS + 0.1%Tween-20 (PBS-T) at room temperature and were incubated overnight at 4 ºC with a pSMAD1/5 (1:500, Ser463/465, Catalog #9516, Cell Signaling). Then, incubation with the secondary antibody goat anti-mouse IgG-HRP [1:2500 (Vol:Vol), Cell Signaling Technology, Danvers, MA, USA, 7074 S] was conducted for 2 h at room temperature. The chemiluminescent signal was digitized using an ImageQuant LAS 500 chemiluminescence CCD camera (GE Healthcare Life Sciences, USA, 29,005,063). The monoclonal anti-β-actin-peroxidase antibody produced in mouse (Sigma-Aldrich Cat# A3854, RRID:AB_262011) was used as the loading control. Membranes were probed sequentially for this purpose, after detection of an antibody membranes were stripped by washing extensively in TBS-T, three times for 10 min each, and repeating the blocking step, and then the membranes are re-probed with anti-β-actin-peroxidase antibody. In all cases, intensities of protein bands (optical density (OD)) were quantified by ImageJ software, and the relative abundance of each protein was normalized to the total–actin expression in the corresponding lane. The ratio of the OD of the protein concerned (pSMAD1/5) in relation to actin is presented in the form of bar charts.

### Statistical analysis

All statistical tests were performed using the software package SigmaStat (Systat Software Inc., San Jose, CA, USA). Cleavage rate, blastocyst yield, number of cells per blastocyst, relative mRNA abundance levels, and intensities of protein bands, were normally distributed with homogeneous variance, so one-way analysis of variance (ANOVA) with arcsine data transformation, followed by Tukey´s test, was performed to evaluate the significance of differences between groups. Values were considered significantly different at *P* < 0.05. Unless otherwise indicated, data are presented as the mean ± s.e.m.

## Results

### MiR-148b mimics can be taken up by bovine embryos

**Fig. 1:**
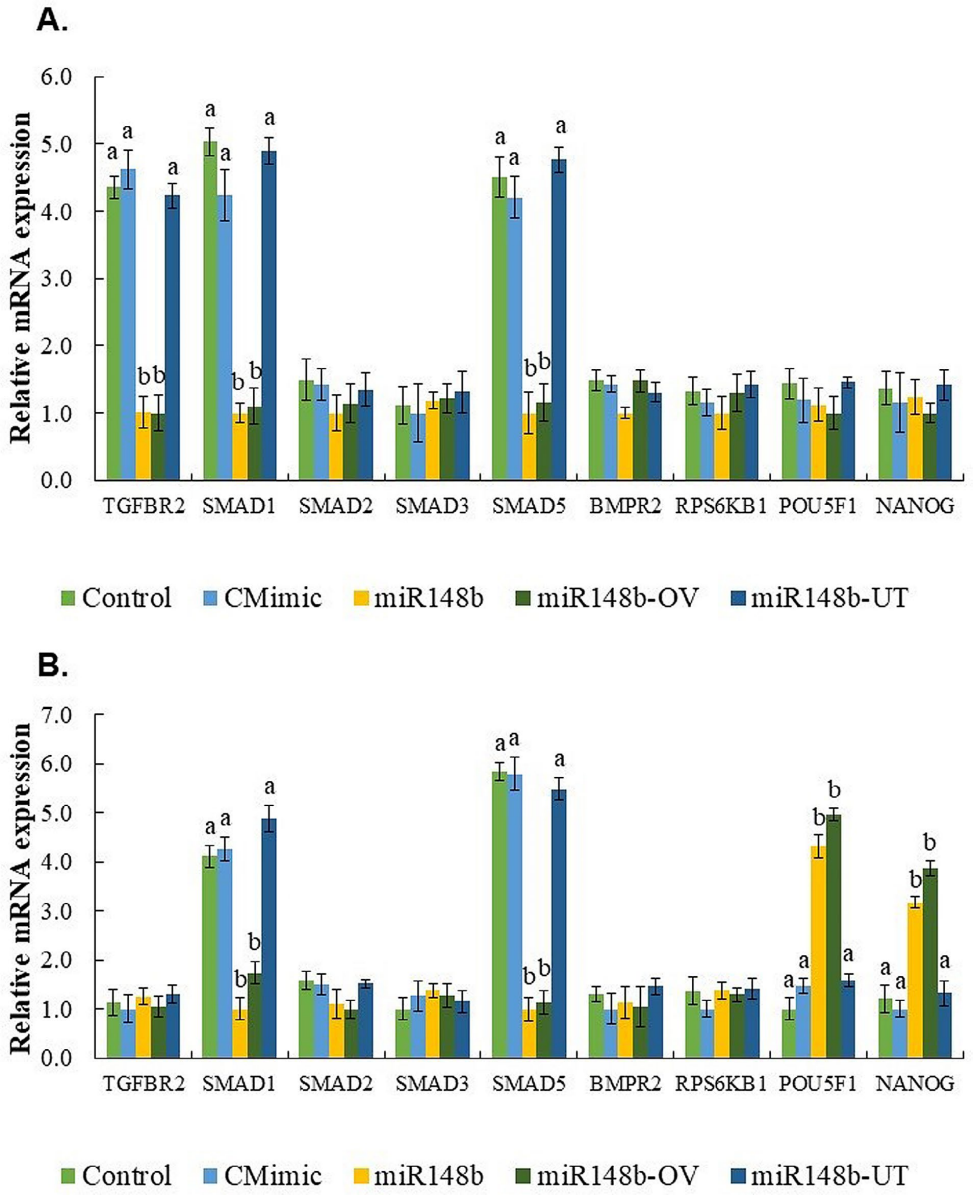
MiR-148b mimics is taken up by bovine embryos and influences on embryo development. (**A**) Representative fluorescence images that illustrate the passage of miRNA mimics through the zona pellucida of 16-cell stage bovine embryos and blastocyst. Presumptive zygotes were cultured from 21 h post-insemination (hpi) until day 8 pi (CMimic) along with commercial triple-RNA-strand mimics prelabeled with 5′ FAM (5-carboxyfluorescein, green) or PBS (Control). Zona-intact bovine embryos at 16-cell stage and blastocyst (5′FAM-labeled or PBS) were washed, fixed, and stained with Hoechst (blue) to visualize the nuclei. The merged images demonstrate uptake of green, fluorescent–labeled mimics by 16-cell stage embryos and blastocyst. (**B**) Relative expression level of miR-148b of bovine embryo cultured from Day 1 until Day 7 pi with or without miR-148b mimics or control mimics. (**C**) Developmental rates of in vitro produced bovine embryos cultured from Day 1 until Day 7 p.i. with or without miR-148b mimics or control mimics. Results are expressed as mean ± s.e.m. Significant differences (*P* < 0.001) are indicated with different letters

Fluorescence staining showed that miR-148b mimics can be taken up from bovine embryos by passive transfection (Gymnosis) (Fig. 1A). In addition, RT-qPCR results showed that miR-148b is indeed taken up by the 16-cell embryos and blastocysts as its levels were noticeably higher (approximately 50 times) in the miR-148b mimics supplemented group compared with control groups (Control and CMimic) (Fig. 1B). No significant differences were found in cleavage or blastocyst rate between the miR-148b mimics group and the control groups (Fig. 1C).

### SMAD5 could be a potential target for miR-148b

**Fig. 2:**
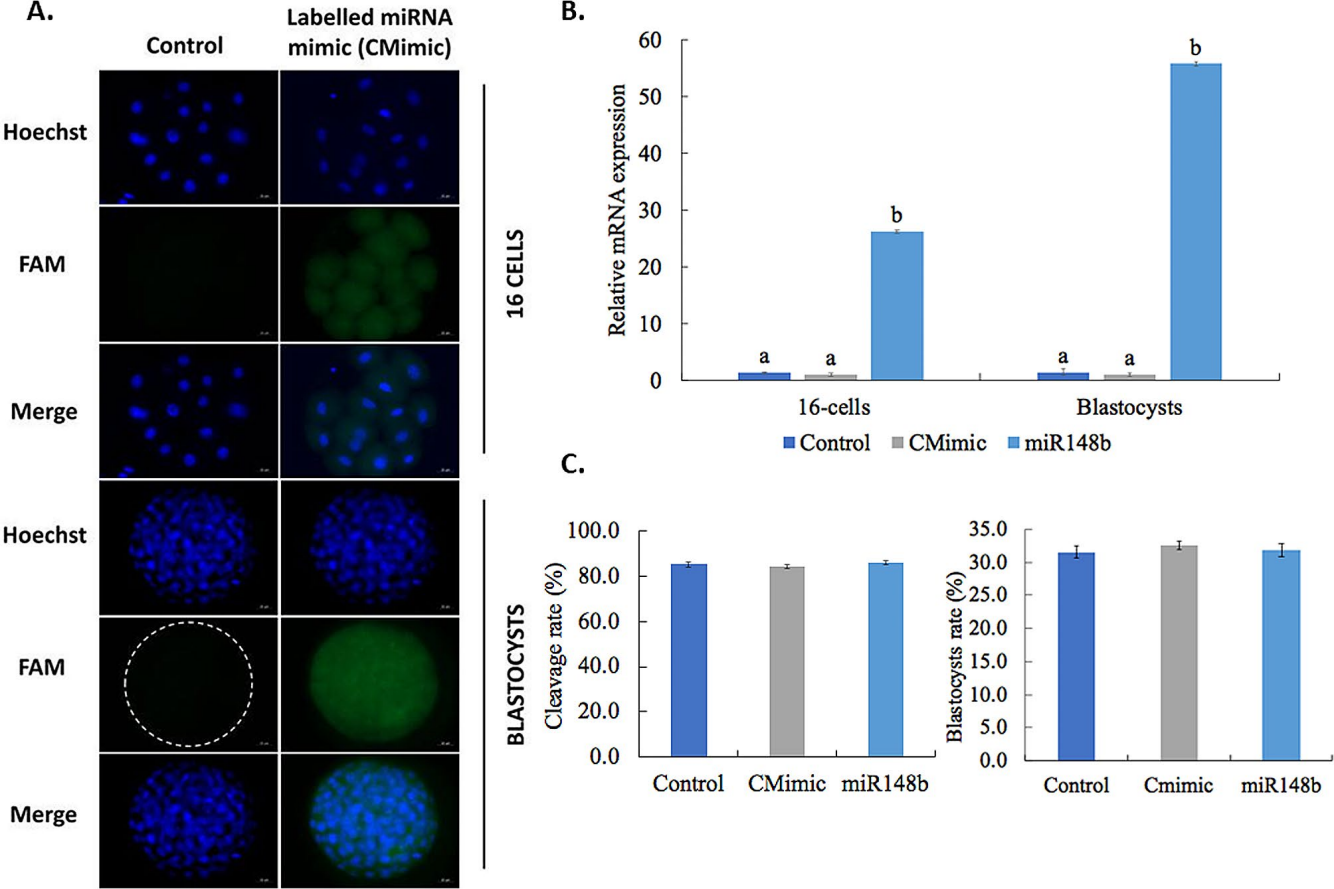
Transcript abundance of target genes in embryos after IVC, with or without miR-148b. Embryos cultured from presumptive zygotes to blastocyst in SOF + 0.3% BSA (Control), supplemented with 1 µM control mimics (CMimic), or supplemented with 1 µM of miR-148b mimics (miR148b). Transforming growth factor-beta 2 receptor (*TGFBR2*); SMAD family member 1 (*SMAD1*); SMAD family member 2 (*SMAD2*); SMAD family member 3 (*SMAD3*); SMAD family member 5 (*SMAD5*); Bone morphogenetic protein receptor 2 (*BMPR2*); Ribosomal Protein S6 Kinase Beta-1 (*RPS6KB1*); POU Class 5 Homeobox 1 (*POU5F1*); Nanog Homeobox (*NANOG*). Data are the mean ± s.e.m. Different letters indicate significant difference (*P* ≤ 0.05) between treatments

Computational algorithms identified *SMAD5*, *LTBP1*, *SKP1*, *PIK3R3*, *GDF6*, *PTEN* as target genes (Supplementary Table S2). These target genes contain a 3′UTR element that is complementary to the miR-148b seed sequence. Real time qPCR analysis of these genes showed that mRNA abundance of *SMAD5* decreased in BD7 from miR-148b group compared to controls. No differences were observed for the other transcripts (Fig. 2).

### **MiR-148b modulates the quality of** in vitro **produced blastocysts**

To determine the effect of miR-148b on embryo development and quality during different times, we supplemented miR-148b mimics to the culture medium of presumptive zygotes and cultured them until day 8 (See 2.7 Section: Experimental design – Experiment 3). Cleavage and blastocyst rates were not affected in any group (Table 2), while supplementation of miR-148b mimics had a beneficial effect on embryo quality. We observed in blastocysts cultured in the presence of miR-148b mimic during the entire culture period ((Day 1 to Day 7- (miR148b)) or from Day 1 to Day 4 ((representing miRNA effect in the oviduct-(miR148b-OV)) the number of total cells, TE and ICM cells was significantly higher than the other groups (*P* ≤ 0.001). Blastocysts from Control, CMimic and miR148b-UT groups did not show differences (Table 3).

**Table 2:** Kinetics of development at 48 and 96 h post-insemination (hpi) and cumulative blastocyst rates on Days 7 and 8 after IVC with or without miR-148b mimic or control mimics

	IVC N	Total cleaved N (%±SEM)	< 16 cells N (%±SEM)	≥ 16 cells N (%±SEM)	IVC 96 hpi	Blastocysts	
						D7	D8
						N (%±SEM)	N (%±SEM)
**Control**	1001	874 (87.3 ± 0.7)	230 (23.2 ± 0.6)	644 (64.1 ± 0.7)	881	212 (24.4 ± 0.5)	274 (31.2 ± 0.6)
**CMimic**	1011	878 (86.9 ± 0.6)	229 (22.7 ± 0.4)	649 (64.2 ± 0.7)	891	212 (23.8 ± 0.5)	272 (30.6 ± 0.8)
**miR148b**	1019	891 (87.5 ± 0.4)	228 (22.5 ± 0.5)	663 (64.9 ± 0.6)	899	217 (24.2 ± 0.6)	282 (31.4 ± 0.8)
**miR148b-OV**	1052	917 (87.7 ± 0.5)	253 (23.1 ± 0.4)	664 (64.5 ± 0.5)	932	223 (24.4 ± 0.5)	290 (31.0 ± 0.5)
**miR148b-UT**	1056	927 (87.4 ± 0.5)	245 (23.3 ± 0.6)	682 (63.1 ± 0.4)	936	228 (23.9 ± 0.6)	291 (31.1 ± 0.8)

Embryos cultured from presumptive zygotes to blastocyst in SOF + 0.3% BSA (Control), SOF + 0.3% BSA supplemented with 1 µM control mimics (CMimic), or SOF + 0.3% BSA supplemented with 1 µM of miR-148b (miR148b) or embryos cultured from presumptive zygotes to 16- cell stage in SOF + 0.3% BSA supplemented with 1 µM of miR-148b (miR148b-OV), or embryos cultured from 16-cell stage to BD7 in SOF + 0.3% BSA supplemented with 1 µM of miR-148b (miR148b-UT). Data are the mean ± s.e.m. Within columns, different superscript letters indicate significant difference (*P* ≤ 0.001) between treatments

**Table 3:** Embryo quality assessment of bovine embryos after IVC with or without miRNA-148b mimic or control mimics

	No. Blastocysts Processed	No. Total nuclei	No. ICM nuclei	No. TE nuclei	Ratio ICM/TE
**Control**	30	108.8 ± 1.0^b^	36.8 ± 1.0^b^	66.2 ± 0.7^b^	0.5 ± 0.02
**CMimic**	30	105.3 ± 1.2^b^	36.8 ± 0.8^b^	68.5 ± 1.1^b^	0.5 ± 0.02
**miR148b**	30	136.4 ± 1.6^a^	43.9 ± 1.3^a^	92.5 ± 0.9^a^	0.5 ± 0.01
**miR148b-OV**	30	135.3 ± 1.5^a^	42.7 ± 0.8^a^	92.6 ± 1.2^a^	0.5 ± 0.01
**miR148b-UT**	30	106.5 ± 1.1^b^	37.5 ± 1.2^b^	69.0 ± 0.9^b^	0.5 ± 0.02

Embryos cultured from presumptive zygotes to blastocyst in SOF + 0.3% BSA (Control), or SOF + 0.3% BSA supplemented with 1 µM control mimics (CMimic), or SOF + 0.3% BSA supplemented with 1 µM of miR-148b (miR148b) or embryos cultured from presumptive zygotes to 16- cell stage in SOF + 0.3% BSA supplemented with 1 µM of miR-148b (miR148b-OV), or embryos cultured from 16-cell stage to blastocyst in SOF + 0.3% BSA supplemented with 1 µM of miR-148b (miR148b-UT). ICM: inner cell mass; TE: trophectoderm. Data are the mean ± s.e.m. Within columns, different superscript letters indicate significant difference (*P* ≤ 0.001) between treatments

**Fig. 3:**
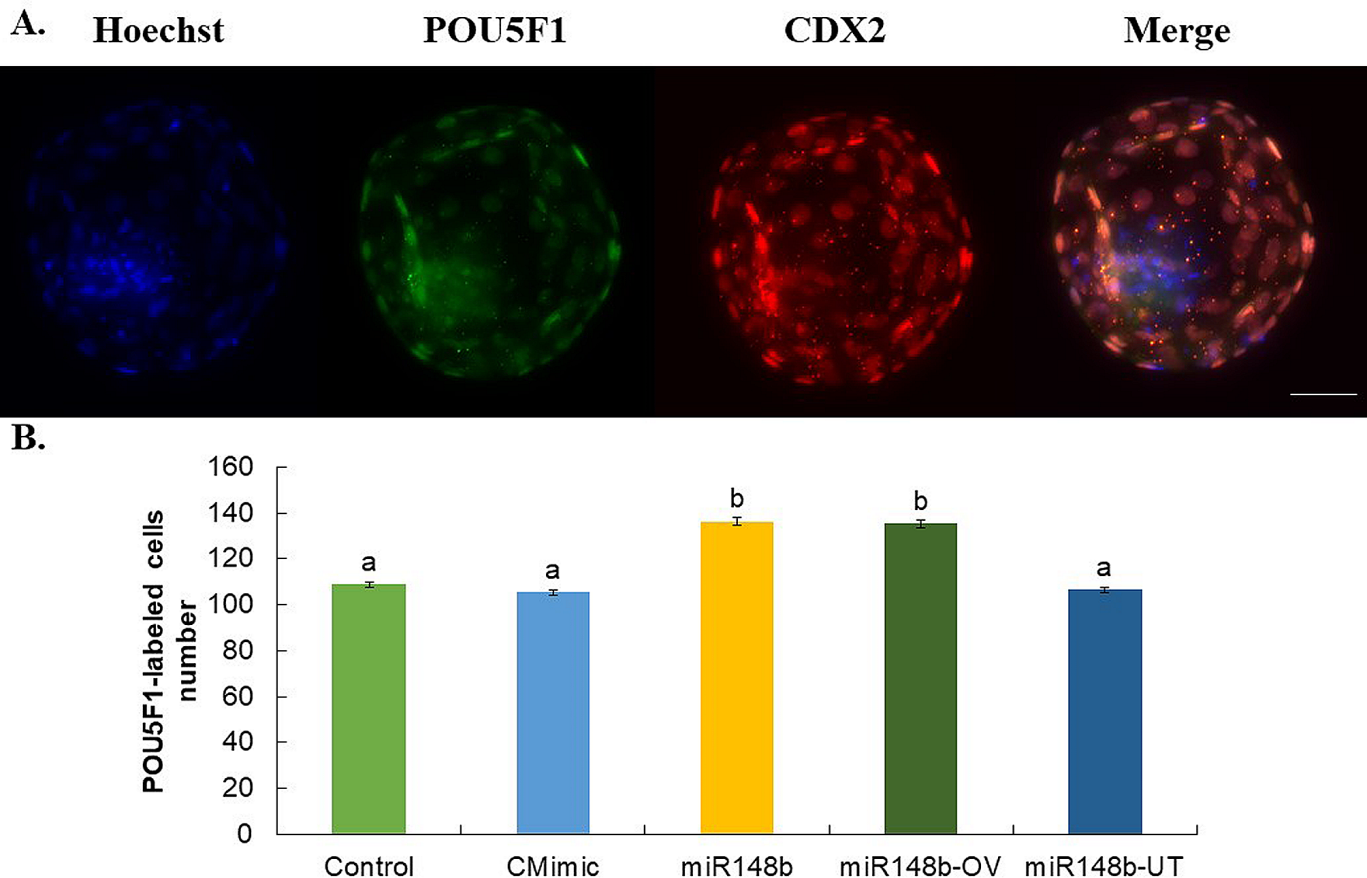
POU5F1 is present in in vitro produced bovine blastocyst. (**A**) Representative fluorescence images of stained with Hoechst (blue - total cell number), POU5F1 (green) and CDX2 (red -trophectoderm cells) in BD7. Scale bar 50 μm. (**B**) Proportion of cells stained positive for POU5F1 markers at Day 7 blastocysts relative to the total cell number. Embryos cultured from presumptive zygotes to blastocyst in SOF + 0.3% BSA (Control), SOF + 0.3% BSA supplemented with 1 µM control mimics (CMimic), or SOF + 0.3% BSA supplemented with 1 µM of miR-148b mimics (miR148b) or embryos cultured from presumptive zygotes to 16- cell stage in SOF + 0.3% BSA supplemented with 1 µM of miR-148b mimics (miR148b-OV), or embryos cultured from 16-cell stage to blastocyst in SOF + 0.3% BSA supplemented with 1 µM of miR-148b mimics (miR148b-UT). Data are the mean ± s.e.m. Different letters indicate significant difference (*P* ≤ 0.05) between treatments

Immunostaining of POU5F1 in blastocysts from all groups, revealed POU5F1 signal in both the ICM as well as in TE cells. The POU5F1 signal was observed in both the cytoplasm and nuclei of these cells (Fig. 3A). Besides, the number of POUF51-labeled cells increased significantly in the miR148b and miR148-OV groups compared to the other groups (Fig. 3B).

### MiR-148b mimics can modulate some transcript of TGF-β signaling pathway

**Fig. 4:**
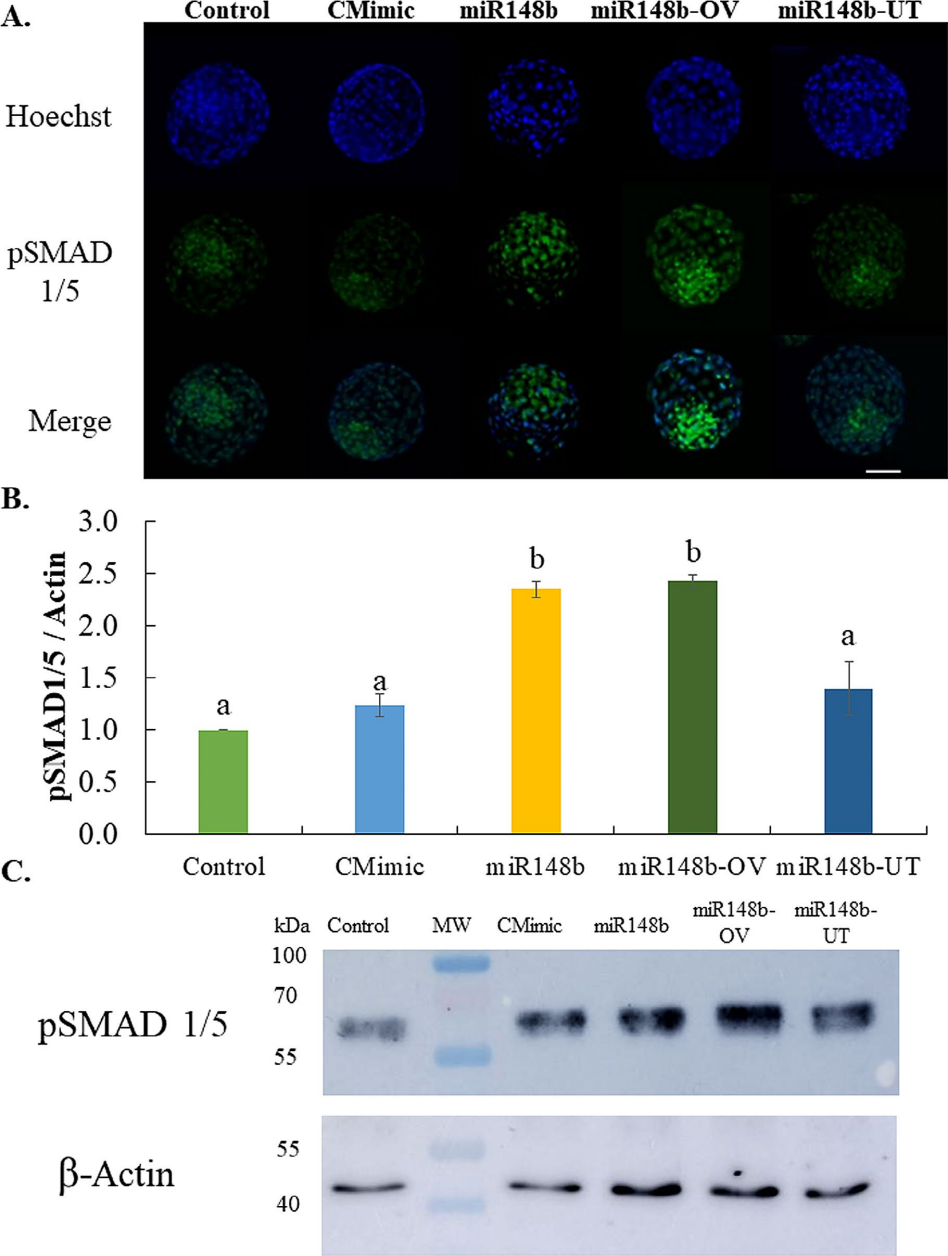
TGF-β pathway genes mRNA in 16-cell embryos and BD7 cultured with or without miR-148b. (A) Relative mRNA abundance in 16-cell stage embryos cultured in SOF + 0.3% BSA (Control), supplemented with 1 µM control mimics (CMimic), or supplemented with 1 µM of miR-148b (miR148b) or embryos cultured from presumptive zygotes to 16- cell stage in SOF + 0.3% BSA supplemented with 1 µM of miR-148b (miR148b-OV), or embryos cultured from 16-cell stage to blastocyst in SOF + 0.3% BSA supplemented with 1 µM of miR-148b (miR148b-UT). (B) Relative mRNA abundance in blastocysts from Control, CMimic, miR148b, miR148b-OV and miR148b-UT experimental groups. Data are the mean ± s.e.m. Different letters indicate significant difference (*P* ≤ 0.001) between treatments

The mRNA transcripts of *SMAD1* and *SMAD5* were significantly decreased (*P* ≤ 0.001) in 16-cell stage embryos (Fig. 4A) and blastocysts (Fig. 4B) from the miR148b and miR148b-OV groups compared to the remaining groups. Additionally, *TGFBR2* mRNA was specifically downregulated in 16-cell embryos from the miR148b and miR148b-OV groups when compared to the other groups. Conversely, *POU5F1* and *NANOG* mRNAs were upregulated in blastocysts (*P* ≤ 0.001) of the miR148b and miR148b-OV groups compared to the other groups. No significant differences were observed for *SMAD2*, *SMAD3*, *BMPR2* and *RPS6KB* between groups.

### MiR-148b mimics regulates the expression of SMAD1/5 protein

**Fig. 5:**
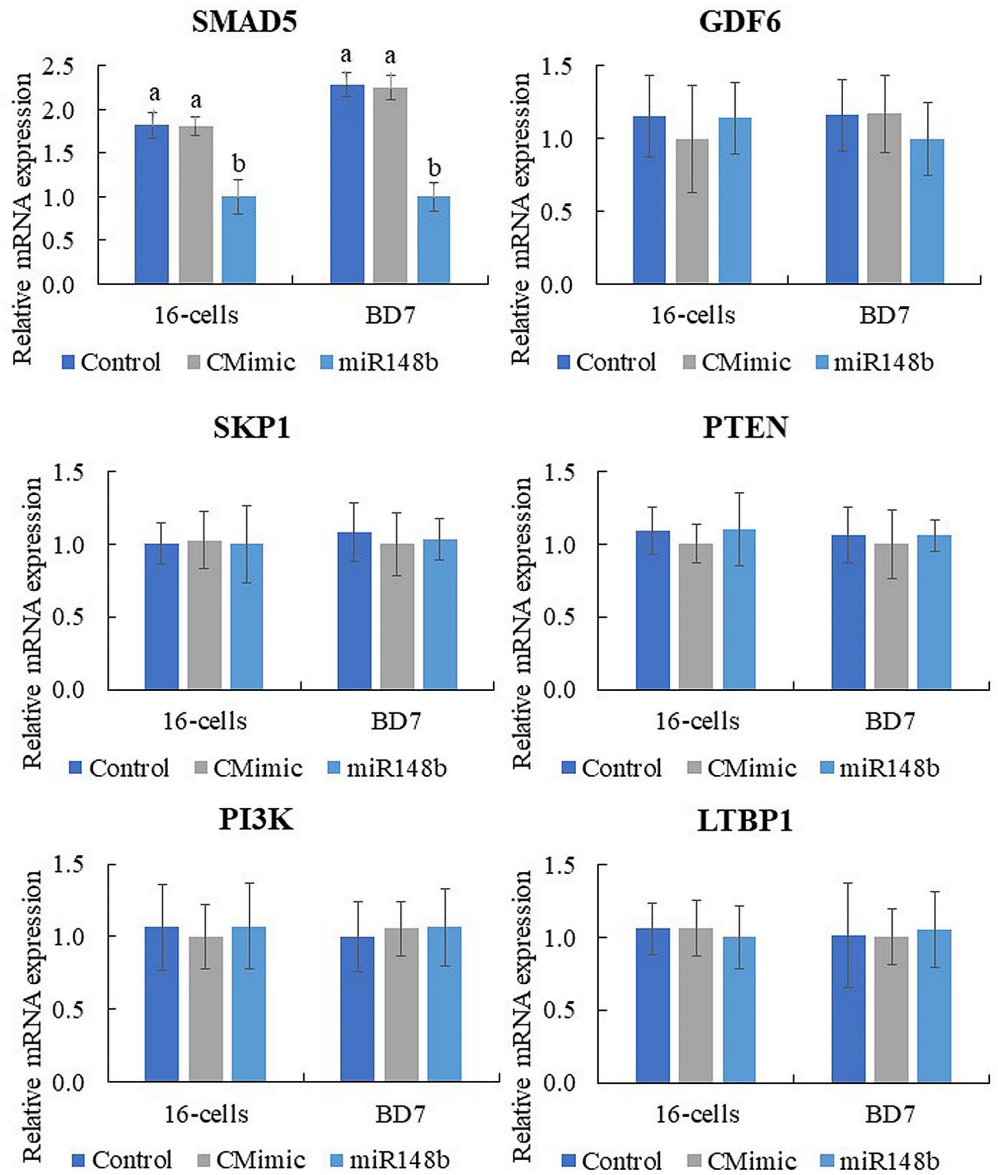
pSMAD1/5 phosphorylation level in blastocysts after IVC with or without miRNA-148b mimic. (**A**) Blastocyst analyzed for localization and abundance of phosphorylated (active) form of SMAD1/5 (pSMAD1/5) transcription factor. pSMAD1/5 Immunostaining (green) counterstained with Hoechst 33,342 for nuclear staining (blue). Embryos cultured from presumptive zygotes to blastocyst in SOF + 0.3% BSA (Control), supplemented with 1 µM control mimics (CMimic), or supplemented with 1 µM of miR-148b (miR148b) or embryos cultured from presumptive zygotes to 16- cell stage in SOF + 0.3% BSA supplemented with 1 µM of miR-148b (miR148b-OV), or embryos cultured from 16-cell stage to blastocyst in SOF + 0.3% BSA supplemented with 1 µM of miR-148b (miR148b-UT). (**B** and **C**) Quantification of phosphorylation levels and representative WB images of pSMAD1/5 signal from each group. WB data were normalized relative to the abundance of β-actin as a loading control and expressed as mean ± SEM. Values with different superscripts indicate statistically significant differences at (*P* ≤ 0.05)

Immunofluorescence analysis revealed immunoreactive proteins for pSMAD1/5 in bovine blastocysts. On Day 7 blastocysts, pSMAD1/5 increased its phosphorylation levels when miR-148b was present in the culture medium during: D1-D7 (miR148b) or D1-D4 (miR148b-OV), while pSMAD1/5 levels were weaker in blastocysts produced from Control, CMimic and miR148b-UT groups (Fig. 5A). Similarly, the western blot analysis showed that pSMAD1/5 phosphorylation levels were significantly higher in blastocysts produced with miR-148b mimic supplementation in the culture medium during: D1-D7 (miR148b) or D1-D4 (miR148b-OV) compared with remained groups (*P* < 0.05) (Fig. 5B and C).

## Discussion

Early embryonic development thrives within the optimal microenvironment provided by the oviduct. In cattle, the embryo spends four days in contact with oviductal epithelial cells and the various components of the oviductal fluid, including EVs. These vesicles are potentially involved in maternal communication with gametes and embryos (reviewed in Cajas et al. [1], as they transfer biomolecules such as miRNAs that can modulate the activities of recipient cells.

Previous studies have demonstrated that in vivo-derived EVs from the bovine oviduct can traverse the zona pellucida (ZP) and be taken up into the cytoplasm of blastocyst cells. This suggests that molecular cargos in oviductal EVs can be incorporated into the developing embryo, as observed in studies by Almiñana et al. [7] and Leal et al. [25]. Furthermore, it has been shown that the addition of oviductal EVs to in vitro culture media enhances embryo quality and blastocyst hatching rate in bovine embryos [7].

Along the same lines, we demonstrated that EVs from bovine oviductal epithelial cell (BOEC) conditioned media can be isolated, characterized, and successfully used for in vitro embryo culture, resulting in improved blastocyst quality [43]. Additionally, supplementation of in vitro culture media with EVs from oviductal fluid during in vitro culture enhances development and increases blastocyst quality in terms of cryotolerance and the expression patterns of quality-related genes [6].

Bauersachs et al. [44] showed that supplementation of in vitro culture media with EVs from oviductal fluid alters the embryonic transcriptome of bovine blastocysts, leading to a decrease in apoptosis of embryonic cells and improved embryo viability. Furthermore, specific miRNAs identified in oviductal EVs can modulate the expression of certain genes. For instance, Sp3 Transcription Factor expression can be targeted by miR-27a-3p, miR-484, miR-1260b, and miR-218-5p; and NANOG can be targeted by miR-34a-5p, miR-34c-5p, miR-34b-3p, miR-335-5p, miR-128-3p, miR-150-5p, and miR-125b-2-3p [44].

Recently, our group demonstrated that mimicking physiological conditions using EVs from OF and UF in sequential IVC does not affect embryo development but significantly improves blastocyst quality. The observed improvements include enhanced survival rates after vitrification/warming, increased total cell number, altered lipid content, and relative changes in the expression of lipid metabolism transcripts and lipase activation. The contents of EVs, particularly miRNAs, may contribute to these observed effects. Notably, our results highlights the increased abundance of microRNA 148b (bta-miR-148b) in EVs derived from oviductal fluid collected during the early luteal phase (Day 1 to 4) in heifers [25]. MiR-148b-3p plays a significant role in cell proliferation, migration, and invasion in human cancer cells, and its effect depends on the level of expression [26, 27]. Moreover, the downregulation of miR-148b-3p in both human and mouse frozen-thawed sperm affects its expression during subsequent embryonic development, resulting in a significantly lower embryo formation rate [45].

Here, we verified that synthetic miR-148b (designed to replicate naturally occurring, mature miRNA), when supplemented to the culture medium, is taken up by embryos of < 16 cells and BD7 through passive transfection. We also validated SMAD5, an essential transcriptional effector of the BMP/TGF-β pathway involved in embryonic development, as a potential target gene for miR-148b. In our functional study, we observed that the presence of miR-148b during the entire culture period (Day 1 to Day 7) or from Day 1 to Day 4 (representing the effect of miR-148b when the embryo is in the oviduct) resulted in an improvement of embryo quality. While embryo development remained unaffected, we noticed an increase in the total number of cells, trophoblast (TE) and inner cell mass (ICM) cells, along with a higher number of cells labeled with POUF51. Moreover, miR-148b exhibited a dual effect by modulating the relative abundance of key genes associated with cellular differentiation and proliferation in the BMP/TGF-β signaling pathway, while also increasing the level of p-SMAD1/5, thus confirming its influence on this pathway. This study suggests that the addition of miR-148b to in vitro culture media enhances embryo quality, indicating its potential significance as a key signaling molecule for embryo-maternal interaction in the oviduct during the preimplantation period. Furthermore, it highlights the involvement of extracellular vesicles in delivering miR-148b.

Besides, our data revealed that fluorescently tagged miRNA mimics can be incorporated by embryos without the addition of transfection reagents. This phenomenon, known as “gymnosis” or passive transfection, is a poorly understood process through which various cell types can internalize miRNAs in vitro without association with protein complexes or the addition of transfection reagents [46, 47]. Previous studies have also reported this uptake mechanism in bovine embryos [5]. Furthermore, we confirmed through qPCR that the miR-148b mimic is indeed taken up by 16-cell embryos and blastocysts, as evidenced by significantly higher levels (approximately 50 times higher) compared to control groups. These findings align with a study by Lin et al. [23], which demonstrated the uptake of miR-10b by embryos, where qPCR results exhibited notably higher levels (approximately 70 times higher) compared to the control group.

Based on computational algorithm analysis, we evaluate the modulation of putative target genes of miR-148b in cattle. The results revealed that the mRNA abundance of SMAD5 decreased in 16-cell embryos and Day 7 blastocysts from the miR-148b group compared to the control group. Hence, our study suggests that miR-148b directly targets SMAD5 and inhibits the transcription of this gene. MiRNAs predominantly function to reduce target mRNA levels [48]. This reduction in mRNA levels is associated with poly(A) tail shortening, suggesting a model in which miRNAs induce mRNA de-adenylation. This de-adenylation process promotes decapping and facilitates more rapid degradation through standard mRNA turnover processes [49–51].

To further comprehend the mechanisms by which miR-148b affects early embryonic development, we designed a functionality test by adding a specific miRNA mimetic (miR-148b) to the embryo culture medium. Our results are consistent with previous studies that have demonstrated that the addition of miRNA mimics to the embryo culture medium does not adversely affect development [5, 23]. In contrast, a recent study by Aoki et al. [52] reported enhanced embryonic development following the supplementation of miRNA mimics in the culture media. This suggests that the efficacy of improving in vitro embryo production might be contingent upon the specific functional role exerted by the added miRNA.

On the contrary, miR-148b exhibited a notable improvement in various embryo quality parameters within the miR-148 (supplemented throughout the entire period of IVC) and miR148-OV (supplemented during days 1 to 4 of IVC, representing the miRNA’s effect in the oviduct) groups. Specifically, it led to a significant increase in the total number of cells, trophoblast (TE), inner cell mass (ICM) cells, and POUF51-labeled cells. Furthermore, miR-148b demonstrated its regulatory role by influencing the expression of key genes involved in cellular proliferation and differentiation, such as *POU5F1* and *NANOG*.

During mammalian preimplantation development, OCT4/POU5F1 plays a critical role as a transcription factor, with diverse functions [53], encompassing the maintenance of pluripotency and the regulation of differentiation events [54]. Our results showed that the pluripotency factor OCT4/POU5F1 is consistently expressed across the entire bovine Day 7 blastocysts, similar to the observations by Simmet et al. [55, 56] and Kirchhof et al. [57] in both in vivo and in vitro produced bovine embryos on days 7, 9, and 12. Furthermore, we observed an increased expression of *POU5F1* and *NANOG* transcripts in blastocysts produced with miR-148b supplemented throughout the entire period of IVC or during days 1 to 4 of IVC to simulate the miRNA effect in the oviduct. Previous studies have indicated that *NANOG* activation does not rely on the embryonic activation of *OCT4* [53, 58]. However, the absence or very low levels of the epiblast marker NANOG in OCT4 knockout (KO) blastocysts on day 7 suggests that the maintenance of epiblast cells during the early stages of the second lineage differentiation fails in the absence of OCT4, thereby confirming the requirement of OCT4/POU5F1 for NANOG expression in bovine blastocysts [55]. In another study by the same group, OCT4 KO blastocysts generated through somatic cell nuclear transfer and zygote injection demonstrated that both epiblast maintenance and hypoblast differentiation rely on OCT4. Consequently, it was concluded that OCT4 is necessary for the maintenance of pluripotency in the epiblast and the differentiation of the hypoblast during the second lineage differentiation in bovine preimplantation embryos [55, 56].

In addition, the pluripotent state in murine and human embryonic stem cells is maintained through the coordinated activity of BMP signaling, along with the transcription factors *OCT4* and *NANOG* [59–61]. BMPs belong to the TGF-β superfamily and employ downstream effectors—*SMAD1, SMAD5*, and *SMAD8*—to transmit signaling. Notably, a fascinating finding suggests that NANOG physically interacts with SMAD1, thereby interfering with the recruitment of coactivators to active SMAD1 complexes and influencing the activity of BMP signaling [62]. Studies provide evidence that bovine embryos can exhibit a response to BMP2 stimulation within the initial 3 days of in vitro culture. This response manifests as an increase in blastocyst mRNA for *NANOG* and *CDX2*, which is measured 4 days later. Furthermore, the effects of BMP2 treatment on cell allocation indices are observed even after the administration of the treatment. The precise mechanisms underlying the heightened expression of blastocyst *NANOG* and *CDX2* mRNA in response to BMP2 stimulation remain unknown. However, previous research has demonstrated that *BMP4* can induce *CDX2* mRNA expression [63] and promoter activity [64] in other cell lines and human ES cells. Notably, both *BMP2* and *BMP4* signaling pathways operate through SMAD1/5 [65]. Collectively, these results reveal the critical role of BMPs in preimplantation embryo development, which opens the possibility of establishing BMP/TGF-β as an important pathway of action for miR-148b in the bovine embryo.

The activity of TGF-β ligands is mediated predominantly through SMAD signaling [66]. Briefly, upon binding between the TGF-β ligand and type II receptor, the type I receptor is recruited and phosphorylated, which in turn activates SMADs (R-SMADs) by phosphorylation. SMAD1/5/8 are activated by BMPs, while SMAD2/3 are activated by activin and TGF-β binding to cognate receptors. Once activated, R-SMADs bind to SMAD4 and are then imported to the nucleus, where they regulate gene transcription [67, 68].

To verify the action of miR-148b on the quality of 16-cell and blastocyst stage embryos, and its possible implication in signaling the BMP/TGF-β pathway, we analyzed the expression of candidate genes like *TGFBR2*, a key regulator of the TGF-β pathway, and *SMAD1, SMAD2, SMAD3, SMAD5, BMPR2, RPS6KB1*. The results showed that the relative abundance of TGFBR2 was only decreased in 16-cell embryos for the miR148 (supplemented throughout the entire period of IVC) and miR148-OV (supplemented during days 1 to 4 of IVC, representing the miRNA effect in the oviduct) groups. Barrera et al. [69] reported that TGFBR2 expression is present from the 2- to 8-cell stage embryos but was not detected at the blastocyst stage. Unfortunately, they did not analyze the embryos at > 16-cell stage, so we speculate that the expression of TGFBR2 tends to decrease during the major phase of embryonic genome activation (MJEGA-8- to 16-cell stage); however, this needs to be verified.

Our findings revealed significant decreases in mRNA transcripts of SMAD1 and SMAD5 in 16-cell embryos and blastocysts from the miR148 (supplemented throughout the entire period of IVC) and miR148-OV (supplemented during days 1 to 4 of IVC, representing the miRNA effect in the oviduct) groups. These results are consistent with a previous study by Lee et al. [67] which reported that SMAD1 mRNA levels remained elevated after fertilization until the 4-cell stage but showed a subsequent decrease in 16-cell, morula, and blastocyst stage embryos. In contrast, SMAD5 mRNA levels increased after fertilization at the pronuclear, 2-cell, and 4-cell stages, reaching their lowest levels at the 16-cell, morula, and blastocyst stages [67]. These findings suggest that SMAD1 and SMAD5 transcripts are of maternal origin, and their downregulation may play a crucial role following major embryonic genome activation.

Moreover, miRNAs are strong candidates for facilitating maternal mRNA degradation during the maternal-to-embryonic transition (MET) due to their ability to regulate gene expression in a temporally and spatially specific manner. Several studies have demonstrated the involvement of miRNAs in the development of various species, particularly in the degradation of maternal transcripts. These studies have identified the presence of miRNAs at distinct developmental stages prior to EGA, and in all cases, miRNA profiles were found to be up-regulated during EGA [35, 50, 70]. These characteristic expression profiles strongly support the hypothesis that miR-148b undergoes processing during EGA to serve as a guide for the degradation of maternal mRNA.

In a previous study [33], we observed the dynamic regulation of ligands, receptors, and signaling components of the BMP signaling pathway during early embryogenesis in bovine. However, the presence of these transcripts does not mean that BMP signaling is active during early embryonic development. To corroborate this, we conducted immunofluorescence analysis to investigate the localization of the pSMAD1/5 protein and performed Western blot analysis to assess its expression in blastocysts. Our findings revealed that pSMAD1/5 protein, produced by BMP-activated receptor kinase, was predominantly localized in the nucleus of Day 7 blastocysts across all groups. This finding is consistent with similar observations made by Rajput et al. [71], suggesting that the activation of SMAD-dependent BMP signaling may occur during embryonic genome activation. This activation could potentially play a role in crucial developmental events such as blastocyst formation and the determination of the first cell lineage in cattle.

Our results indicated that the downregulation of SMAD5 and SMAD1 mRNA could compromise the protein levels. Surprisingly, Western blot analysis demonstrated a higher abundance of p-SMAD1/5 in Day 7 blastocysts cultured with miR-148 throughout the entire in vitro culture period (miR148) or from Day 1 to Day 4 of in vitro culture (representing the miRNA effect in the oviduct, miR148-OV). Interestingly, previous studies have reported a decrease in mRNA abundance for SMAD1 and SMAD5 in bovine blastocysts from the 16-cell stage until the blastocyst stage [68], while another study revealed a significant increase in p-SMAD1/5 from the 8–16-cell stage, persisting through Day 7 blastocysts [71]. These findings align with our observations, suggesting a potential explanation for our results. It is plausible that an alternate biological pathway is activated to promote the phosphorylation of SMAD1/5 as a compensatory mechanism. For instance, previous research has shown that BMP2 stimulation induces the phosphorylation of SMAD1/5 in cancer cells [72]. Nonetheless, this hypothesis requires further investigation.

These findings collectively suggest a potential role of miRNA-148b regulating cell proliferation and differentiation in IVP embryos. The importance of miRNAs in maintaining normal organism function raises the possibility that disruptions in maternal miRNA function could contribute to suboptimal embryo development, affecting both natural fertilization and in vitro fertilization programs. However, it is essential to acknowledge that research in this domain faces limitations due to existing technical challenges.

In conclusion, this study demonstrated that miR-148b is taken up by 16-cell embryos and blastocysts when supplemented in the culture medium. This allowed us to assess the effects of miR-148b supplementation in the IVC medium at different time frames, representing its effects in the oviduct or uterus, on embryo quality. We hypothesized that miR-148b could enhance embryo quality and modify the abundance of key genes involved in cell proliferation and differentiation in IVP embryos. These positive responses of miR-148b on embryonic quality in blastocysts produced in vitro could be modulated by BMP/TGF-β through SMAD5 as a possible target gene for miR-148b. Moreover, our results highlight the importance of providing a more comprehensive understanding of the role of the oviduct during early embryogenesis and the potential role of miR-148b in embryo-oviduct interaction.

### Electronic supplementary material

Below is the link to the electronic supplementary material.

Supplementary Material 1

Supplementary Material 2

## Data Availability

All data generated or analyzed during this study are included in this published article.

## Abbreviations

BD7: Blastocysts Day 7
BMPR2: Bone morphogenetic protein receptor 2
BSA: Bovine serum albumin
EVs: Extracellular vesicles
FCS: Fetal calf serum
ICM: Inner cell mass
IVC: In vitro culture
IVF: In vitro fertilization
IVP: In vitro embryo production
miRNAs: microRNAs
NANOG: Nanog Homeobox
OF: Oviductal fluid
POU5F1: POU Class 5 Homeobox 1
RPS6KB1: Ribosomal Protein S6 Kinase Beta-1
SMAD1: SMAD family member 1
SMAD2: SMAD family member 2
SMAD3: SMAD family member 3
SMAD5: SMAD family member 5
SOF: Synthetic oviductal fluid
TCN: Total cell number
TE: Trophectoderm
TGFBR2: Transforming growth factor-beta 2 receptor
UF: Uterine fluid
