# Supplementary material for: MicroRNA-148b secreted by bovine oviductal extracellular vesicles enhance embryo quality through BPM/TGF-beta pathway
Source: Biol Res. 2024 Mar 23;57:11. doi: 10.1186/s40659-024-00488-z (PMC10960404; doi:10.1186/s40659-024-00488-z)
Supplement: Supplementary file 1 — Supplementary Material 1 [file 40659_2024_488_MOESM1_ESM.docx]

Supplementary Table S1. Details of primers used for RT-PCR analysis

| **Gene** | **Gene name** | **Primer sequence (5’- 3’)** | **Fragment size (bp)** | **GenBank accession number** |
| --- | --- | --- | --- | --- |
| ***BMPR2*** | Bone morphogenetic protein receptor 2 | TCTTTCAGCCACCAATGTCCT  GTTCAGTGGAAATGACCCAGG | 158 | NM_001304285.1 |
| ***GDF6*** | Growth differentiation factor 6 | GGACTTACTCCATCGCCGAG  GAGGAGTGTGCGAGAGATCG | 120 | [NM_001001140.1](https://www.ncbi.nlm.nih.gov/nuccore/NM_001001140.1) |
| ***H2AFZ*** | H2A histone family, member Z | AGGACGACTAGCCATGGACGTGTG  CCACCACCAGCAATTGTAGCCTTG | 209 | NM_174809 |
| ***LTBP1*** | Latent Transforming Growth Factor Beta Binding Protein 1 | TCATCTGCATCCACAGTTTCCA  GTGTTGATCCTGAGCGCATTC | 277 | [NM_001103091.1](https://www.ncbi.nlm.nih.gov/entrez/viewer.fcgi?db=nucleotide&id=156718111) |
| ***NANOG*** | Nanog homeobox | TGTTCTCGCAGACCCAGCTGTG  TTCCAGCTCTGGTTGCTCCACG | 331 | NM_001025344 |
| ***PIK3R3*** | Phosphoinositide-3-kinase regulatory subunit 3 | TCCACCAAAGCCACCTAAGCC  CTCCCTTCCGCAAGGTCAAAG | 212 | NM_174796 |
| ***POU5F1*** | POU class 5 homeobox 1 (Oct3/4) | CGAGTATCGAGAACCGAGTG  CAGGGTTCTCTCCCTAGCTC | 440 | NM_174580.1 |
| ***PTEN*** | Phosphatase and tensin homolog | TCCAGAGCCATTTCCATCCTG  GCTTTGAATCCAAAAACCTTACTAC | 274 | NM_001319898.1 |
| ***RN18S1*** | 18S ribosomal RNA | AGAAACGGCTACCACATCCAA  CCTGTATTGTTATTTTTCGTCACTACCT | 90 | [NR_036642.1](https://www.ncbi.nlm.nih.gov/nuccore/NR_036642.1) |
| ***RPS6KB1*** | Ribosomal Protein S6 Kinase Beta-1 | GGGATAGAGCAGATGGACGTG  TGGTCGTTTGGAGATCATGGG | 117 | NM_205816.1 |
| ***SKP1*** | S-phase kinase-associated protein 1 | GCCATCTCCTTGAGCCCTAC  CATTTGGCAAGGGGACTGGA | 171 | NM_001034781.2 |
| ***SMAD1*** | SMAD family member 1 | TGTGAACCACGGCTTCGAGACG  TCCTGGCGGTGGTATTCTGCTC | 100 | NM_001076223.2 |
| ***SMAD2*** | SMAD family member 2 | TGCCGAGTGCCTAAGTGACA  GGTGCCAGCCATATCTCTGATT | 71 | NM_001046218 |
| ***SMAD3*** | SMAD family member 3 | GGAGCCGAGTACAGGAGACA  AAAGGTCCATTCAGGTGCAG | 81 | [NM_001205805](https://www.ncbi.nlm.nih.gov/nuccore/NM_001205805) |
| ***SMAD5*** | SMAD family member 5 | TTGCTCAGCTTCTGGCTCAGTC  TTGCCGGTGATACTCTGCTCC | 120 | NM_001077107.3 |
| ***TGFBR2*** | Transforming growth factor beta 2 receptor | CTCCGTTCGGGTCTAAGGTG  GGTCATGGTCCCAGCATTCG | 159 | NM_001159566 |

Supplementary Table S2. Forecast of miRNA-148b for gene targets.

|  | **Predicted consequential pairing of target region (top) and miRNA (bottom)** | **Site type** | **Conserved branch length** | **Context**  **++ score** | **context++ score percentile** | **PCT** |
| --- | --- | --- | --- | --- | --- | --- |
| Position 51-57 of **SKP1** 3' UTR  [bta-miR-148b](http://www.mirbase.org/cgi-bin/mirna_entry.pl?acc=bta-miR-148b) | 5'  ...AUUCCAAAUACUAGUUGCACUGC                       ǀ \| \|\| \|\| \|  3'      UGUUUCAAGACACUACGUGACU | 7mer-m8 | 9.943 | -0.48 | 98 | 0.92 |
| Position 532-538 of **SMAD5** 3' UTR  [bta-miR-148b](http://www.mirbase.org/cgi-bin/mirna_entry.pl?acc=bta-miR-148b) | 5'  ...UUUGAUGGAACACUAUGCACUGC  \| \|\| \|\| \|\|  3'       UGUUUCAAGACACUACGUGACU | 7mer-m8 | 3.111 | -0.21 | 85 | 0.82 |
| Position 1774-1780 of **GDF6** 3' UTR  [bta-miR-148b](http://www.mirbase.org/cgi-bin/mirna_entry.pl?acc=bta-miR-148b) | 5' ...CACAUUUGCUUUGGAUGCACUGU  \| \|\| \| \|\| \|  3' UGUUUCAAGACACUACGUGACU | 7mer-m8 | 7.635 | -0.28 | 91 | 0.89 |
| Position 35-41 of **LTBP1** 3' UTR  [bta-miR-148b](http://www.mirbase.org/cgi-bin/mirna_entry.pl?acc=bta-miR-148b) | 5'...UAAGCCUGUAUACUCUGCACUGU                  \|\| \|\| \|\| \|  3'    UGUUUCAAGACACUACGUGACU | 7mer-m8 | 4.655 | -0.26 | 90 | 0.62 |
| Position 191-198 of **PIK3R3** 3' UTR  [bta-miR-148b](http://www.mirbase.org/cgi-bin/mirna_entry.pl?acc=bta-miR-148b) | 5' ...ACUCAGCCGUGCCACUGCACUGA  \| \| \|\| \|\| \|  3'      UGUUUCAAGACACUACGUGACU | 8mer | 3.233 | -0.20 | 83 | 0.6 |
| Position 2254-2260 of **PTEN** 3' UTR  [bta-miR-148b](http://www.mirbase.org/cgi-bin/mirna_entry.pl?acc=bta-miR-148b) | 5'  ...AUUAUAAUGGGCUUUUGCACUGU  \|\| \| \|\| \|\|  3'       UGUUUCAAGACACUACGUGACU | 7mer-m8 | 6.839 | -0.30 | 93 | 0.86 |

The concrete miRNA’s parameters for gene targets are as below: site type represents an exact match to positions 2-8 of the mature miRNA (the seed + position 8). The context ++ score (CS) for a specific site is the sum of the contribution of 14 features. The context ++ score percentile rank is the percentage of sites for this miRNA with a less favorable context ++ score. The conserved branch length score is the sum of phylogenetic branch lengths between species that contain a site. PCT, the probability of conserved targeting has been calculated for all highly conserved miRNA families.
