# Supplementary figures and images for: MicroRNA-148b secreted by bovine oviductal extracellular vesicles enhance embryo quality through BPM/TGF-beta pathway
Source: Biol Res. 2024 Mar 23;57:11. doi: 10.1186/s40659-024-00488-z (PMC10960404; doi:10.1186/s40659-024-00488-z)

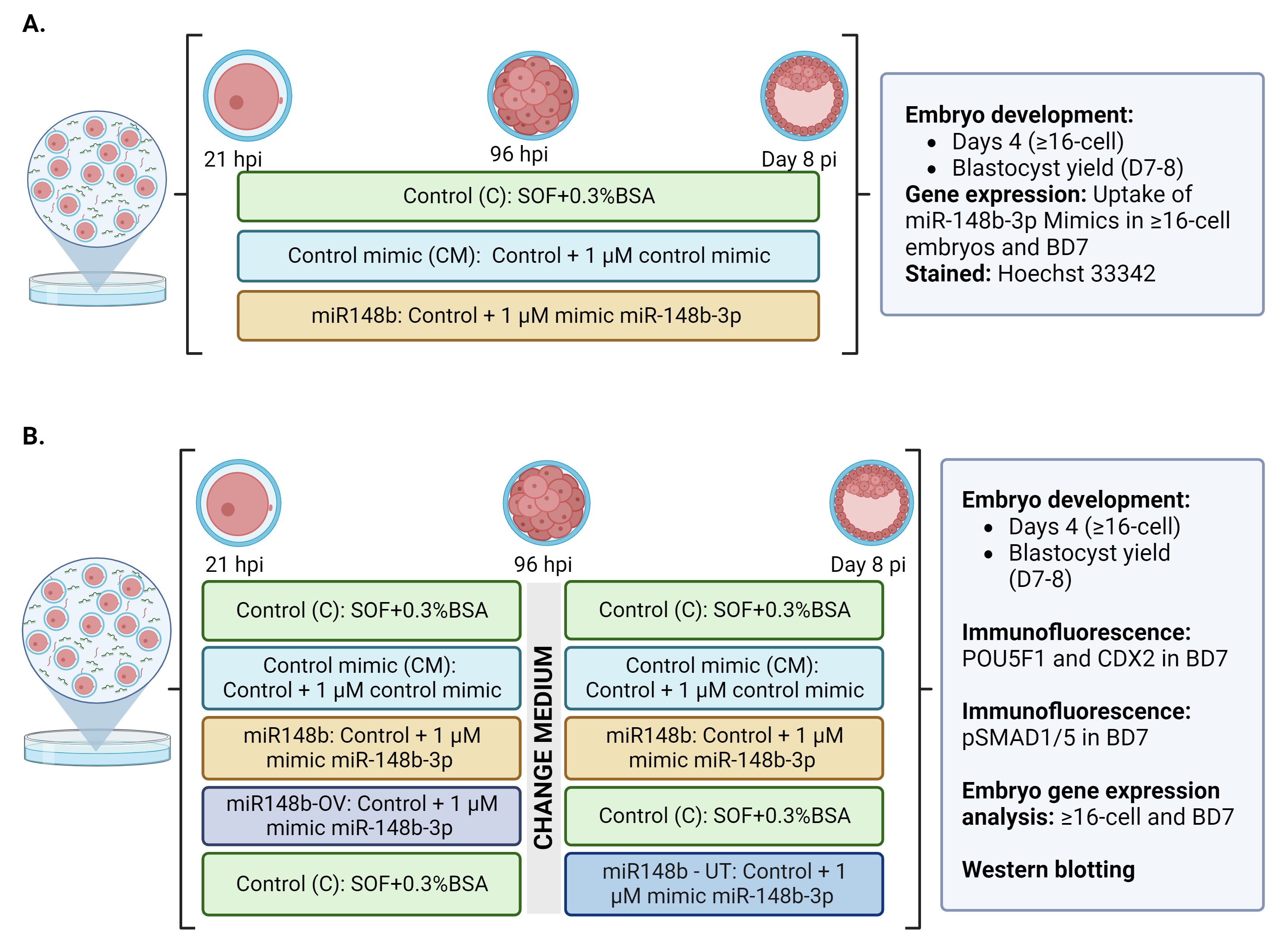

Supplement: Supplementary file 2 — Supplementary Material 2 [file 40659_2024_488_MOESM2_ESM.jpg]
